# Supplementary figures and images for: Longitudinal association between psychological distress and mask-wearing post COVID-19 among psychiatric outpatients in Japan
Source: PLoS One. 2025 Aug 8;20(8):e0329644. doi: 10.1371/journal.pone.0329644 (PMC12334027; doi:10.1371/journal.pone.0329644)

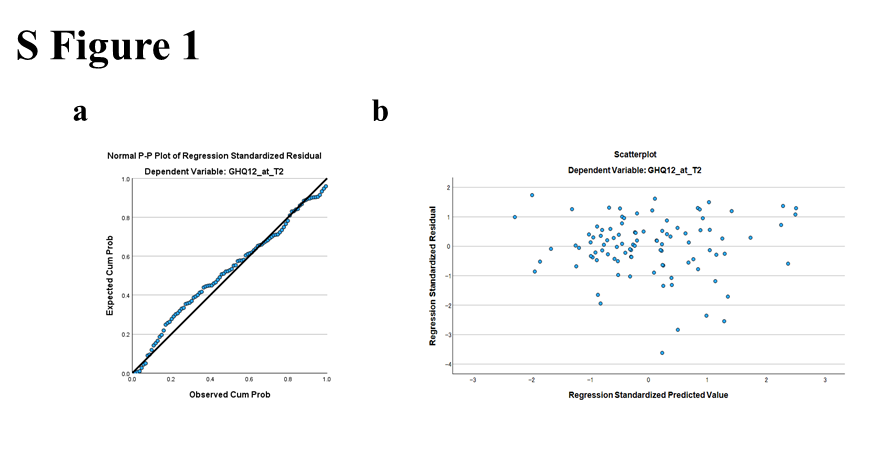

Supplement: S1 Fig — (TIF) [file pone.0329644.s002.tif]
