# Supplementary material for: Longitudinal association between psychological distress and mask-wearing post COVID-19 among psychiatric outpatients in Japan
Source: PLoS One. 2025 Aug 8;20(8):e0329644. doi: 10.1371/journal.pone.0329644 (PMC12334027; doi:10.1371/journal.pone.0329644)
Supplement: Attachment — Submitted filename: Response_to_Reviwers_final.docx [file pone.0329644.s003.docx]

July 5, 2025

**Ref: PONE-D-24-41871**

**Title: Longitudinal association between psychological distress and mask-wearing post COVID-19 among psychiatric outpatients in Japan**

Dr. Bimala Panthee

Academic Editor

*PLOS ONE*

Dear Dr. Bimala Panthee:

Thank you very much for your responses and for providing the reviewers’ comments. We are pleased to have the opportunity to submit a revised version of our manuscript for publication in *PLOS ONE,* titled “**Longitudinal association between psychological distress and mask-wearing post COVID-19 among psychiatric outpatients in Japan**.” The manuscript ID is **PONE-D-24-41871**.

In accordance with the journal's request, we have attached the full review report at the end of this rebuttal letter. We also used a professional English proofreading service (Editage) and made several minor revisions to improve the clarity and readability of the manuscript. In response to the concerns raised about anonymization in S1 Table, we have deleted any potentially identifying information to ensure full compliance with data privacy requirements.

Our point-by-point responses to all comments are as follows:

**Reviewer #1’s comments (***italicized***) and our responses:**

We wish to express our appreciation to the Reviewer for the insightful comments, which have helped us significantly to improve the paper.

**Specific Questions**

**Comment #1-1:** *The Shapiro-Wilk test checks if the data are normally distributed, but it doesn't mention other important values, such as skewness or kurtosis. Non-parametric tests (Wilcoxon) solve this problem, but it would be better to include these indicators.*

**Response #1-1:** Thank you very much for your valuable feedback. In response to your comments, we assessed the normality of the data using the Shapiro–Wilk test, as well as by evaluating skewness and kurtosis. These additional indicators confirmed that the data did not fully meet the assumption of normality. Accordingly, we have revised the Methods section (line 173-183) to justify the use of non-parametric analysis based on these findings.

“Differences in psychological distress before and after the changed mask-wearing guideline were analyzed using the Wilcoxon signed-rank test since the assumption of normality was not fully met. ~~This test was chosen because it is appropriate for comparing two related samples when the assumption of normality, assessed using the Shapiro-Wilk test, is not met.~~ Normality of GHQ-12 scores was assessed using the Shapiro–Wilk test, which showed no significant deviation at T1 (W = 0.979, p = 0.078) but a significant deviation at T2 (W = 0.966, p = 0.007). Skewness and kurtosis values also indicated a roughly normal distribution at T1 (skewness = 0.390, kurtosis = 0.017) and mild non-normality at T2 (skewness = 0.625, kurtosis = 0.798), further supporting the use of a non-parametric test for comparing T1 and T2 scores.”

**Comment #1-2:** *Residual plots or tests for heteroscedasticity, multicollinearity (VIFs), or outliers were not discussed. These are critical for validating regression assumptions.*

**Response #1-2:** Thank you for your insightful comment regarding the validation of regression assumptions. In response, we have conducted additional diagnostic tests to assess heteroscedasticity, multicollinearity, and outliers. Specifically, we visually inspected residual plots to check for heteroscedasticity and found no apparent patterns suggesting non-constant variance. In addition, we calculated the variance inflation factor (VIF) for all predictor variables, which yielded values below 1.5, indicating no significant multicollinearity. We also examined standardized residuals and identified no extreme outliers that would unduly influence the results. In addition, we verified that no outliers exceeded ±3 standard deviations, and this has also been explicitly stated in the manuscript. These diagnostic results have been added to the revised manuscript (Results section, lines 236–242) to ensure transparency and strengthen the validity of the regression analysis.

“To assess whether the assumptions of the regression analysis were met, we conducted several diagnostic tests. Heteroscedasticity was examined by visually inspecting the residual plots, which showed no clear pattern suggesting non-constant variance (S Figure 1). The variance inflation factor (VIF) was also calculated for each independent variable. All VIF values were below 1.5, indicating no serious multicollinearity. In addition, we checked standardized residuals to identify potential outliers. No values exceeded ±3 standard deviations.”

**“S Figure. P–P and Q–Q plots for assessing normality of residuals”**

| Table 2. Multiple linear regression analysis for psychological distress (GHQ-12 scores) | | | | | | | |
| --- | --- | --- | --- | --- | --- | --- | --- |
| Variables | *β* | t | *p* | 95%CI | | | VIF |
| **Mask wearing behavior** | **2.717** | **2.892** | **0.005** | **0.851** | - | **4.584** | **1.047** |
| Age | -0.034 | -1.088 | 0.279 | -0.096 | - | 0.028 | 1.074 |
| Gender | 1.078 | 1.133 | 0.26 | -0.812 | - | 2.967 | 1.098 |
| GHQ-12 at pre-guideline change | 0.779 | 12.701 | <0.001 | 0.657 | - | 0.901 | 1.047 |
| Comparison of those who continued to wear masks and those who stopped wearing it | | | | | | | |
| GHQ-12: General Health Questionnaire-12, CI: confidence interval, VIF: variance inflation factor | | | | | | | |

**Comment #1-3:** *The study examined multiple outcomes (mask wear in different settings: outdoors, shopping, hospitals). If you run multiple tests without correcting for them (like using Bonferroni correction), there is a chance that the number of false positives (Type I errors) will increase.*

**Response #1-3:** Thank you for pointing out this important issue. In this study, mask-wearing behavior was assessed in three different situations (outdoors, while shopping, and in hospitals). To limit the increased risk of Type I error due to multiple comparisons, a Bonferroni correction was applied, adjusting the significance threshold to α < 0.017. The Methods (Methods section, lines 180–183) and Results sections (Results section, lines 202–203) were revised accordingly. The significant findings remained unchanged even after the correction, and the overall interpretation of the results was not affected.

“To account for multiple hypothesis testing across the three situational conditions (outdoors, while shopping, and in hospitals), the Bonferroni correction was applied. The overall alpha level of 0.05 was divided by the number of comparisons, resulting in an adjusted threshold of α = 0.017.”

“After applying the Bonferroni correction for multiple comparisons, ~~A~~ a significant decrease in psychological distress, as assessed by the GHQ-12, was observed after the implementation of the government's mask-wearing mitigation guideline (Fig 2a).”

**Comment #1-4:** *Subgroup analyses by diagnosis (e.g., mood vs. anxiety disorders) were not performed. It may be due to sample size constraints. So you can include it as recommendation for future studies could explore these.*

**Response #1-4:** Thank you very much for your valuable advice. As you pointed out, due to the limited sample size in this study, we were unable to perform subgroup analyses by diagnosis. However, we fully agree that differences between diagnostic groups represent an important issue that should be addressed in future comparative research. In line with your suggestion, we have clearly stated this point in the Discussion section (line 374-377) of the manuscript.

“Lastly, the relatively small sample size limited our ability to conduct subgroup analyses by diagnosis or to examine long-term effects. Future multi-center studies with larger and more diverse populations, as well as longitudinal designs, are needed to confirm and expand upon these findings.”

**Comment #1-5:** *So, Clarify normality test results or justify non-parametric choices more explicitly.
Report model diagnostics (e.g., residuals, VIFs) for regression.
Address potential multiple testing issues if secondary analyses were exploratory.*

**Response #1-5:** We sincerely appreciate your clear summary of the points requiring revision in this paper. We have addressed these issues in Responses #1-1, 1-2, 1-3, and 1-4. We believe that all of your concerns have been adequately addressed; however, please do not hesitate to let us know if any further clarification is needed.

**Reviewer #2’s comments (*italicized*) and our responses:**

We wish to express our appreciation to the Reviewer for the insightful comments, which have helped us significantly improve the paper.

**Specific Questions**

**Comment #2-1:** *Introduction: Your study raises an important discussion about the potential distress associated with mask usage in vulnerable populations. However, one key area that could benefit from further elaboration is the justification for why mask-wearing leads to psychological distress specifically among psychiatric patients.*

**Response #2-1:** Thank you very much for this important and constructive comment. We agree that it is necessary to further clarify why mask-wearing may cause psychological distress, particularly in individuals with mental disorders. In response, we have added a more detailed explanation to the Introduction section (lines 74–84). Specifically, we noted that the physical discomfort associated with mask-wearing (e.g., difficulty breathing, ear pain, and headaches) may affect emotional states and contribute to psychological distress. In addition, we explained that while mask-wearing may provide a sense of protection, individuals with mental disorders might be especially vulnerable to stress due to heightened anxiety about infection and increased sensitivity to how others perceive their behavior.

“Wearing a mask has been shown to cause physical discomfort, such as difficulty breathing, ear pain, and headaches [12], which may influence emotional states and lead to psychological distress. At the same time, wearing a mask may provide a sense of protection, which can help reduce psychological stress [13]. During the COVID-19 pandemic, mask-wearing was recommended to prevent infection. People were expected to adjust their behavior depending on various conditions, including whether they lived in urban or rural areas, the infection status in their surroundings, and whether they were indoors or outdoors. Amid the intense media coverage, vulnerable individuals such as those with mental disorders may have experienced increased psychological stress due to heightened anxiety and greater sensitivity to how others perceived their mask-wearing behavior.”

**“**12.	Gyapong F, Debrah E, Oforiwaa M, Isawumi A, Mosi L. Challenges and Adverse Effects of Wearing Face Masks in the COVID-19 Era. Challenges. 2022;13:67.

13.	Razpurker-Apfeld I, Tal-Or N. Masked distress: The mediated effects of face masks on physical and emotional suffocation. International Journal of Psychology. 2024;59:812-821. **”**

**Comment #2-2:** *Line 102-106 appears misplaced.*

**Response #2-2:** Thank you for pointing this out. As you rightly pointed out, the content in question was not appropriate for the Introduction section. In response to your suggestion, we have removed this content from the Introduction and made the necessary revisions.

“~~Results suggest that the mask-wearing guidance changed after the COVID-19 pandemic may have altered mask-wearing behavior among psychiatric patients and improved their psychological distress. This study provides new insights into the psychological implications of mask-wearing policies in the post-COVID-19 society and informs strategies to support mental health in future public health crises.~~”

**Comment #2-3:** *Results: Line198-200 appears repetitive (data provided in the table)*

**Response #2-3:** Thank you for your comment. We carefully deleted the redundant parts as suggested (lines 219–221).

“Fig 2. Comparison of GHQ-12 total scores pre- and post- guideline change in the mask-wearing behavior. ~~(a) All groups combined;~~ "Others" refers to those who were not wearing masks before the relaxation of the mask-wearing guidelines. ~~(b) Group that continued wearing masks after the relaxation of the mask-wearing guidelines. (c) Group that stopped wearing masks after the relaxation of the mask-wearing guidelines.~~”

**Comment #2-4:** *Discussion: Your findings highlight an important issue, but I believe the discussion could benefit from a stronger emphasis on the clinical and practical implications of your study for psychiatric care. Currently, while the results are well presented, there is limited exploration of how these findings could inform mental health interventions, policy adjustments, or clinical practices.*

**Response #2-4:** Thank you very much for this valuable comment. We fully agree that a clearer discussion of the clinical and practical implications of our findings is essential, especially with regard to psychiatric care in vulnerable populations. In response, we have revised the final part of the Discussion section (lines 327–333) to highlight how changes in public health policy may influence the mental health of individuals living in rural areas, who often face limited access to information and greater social conformity pressures. We also emphasized the importance of culturally and contextually sensitive care, and noted that mental health professionals in such regions should be aware of these factors when supporting patients. We believe that these additions better connect our findings with real-world mental health care and policy planning.

“The results of this study suggest that changes in policy guidelines may also affect the mental health of vulnerable individuals residing in rural areas. Policies are typically formulated with higher consideration of urban residents, but the impact on vulnerable groups in rural areas—who often have limited access to information and tend to hold conservative attitudes—should not be overlooked. Furthermore, healthcare professionals working in mental health care in these regions must understand the psychological care needs of these individuals, which is essential for providing appropriate psychological care to those with mental illnesses. ~~Since social norms may sometimes induce excessive adaptive behavior in a group, resulting in psychological distress at times, it is important for the government to provide appropriate guidelines in line with the local cultural context, with attention to improving mental health and well-being.~~”

**Additional Editor Comments (*italicized*) and our responses:**

We sincerely appreciate your valuable editorial comments. Your feedback has been helpful in improving the clarity and quality of our manuscript.

**Specific Questions**

**Comment #3-1:** *Please incorporate all the comments raised by our reviewers.*

**Response #3-1:** Thank you very much for your comment. We have carefully addressed all comments raised by the reviewers, as outlined in our point-by-point responses above.

**Comment #3-2:** *Further, justification of the study in introduction section needs more elaboration and validity and reliability of the study measures needs to be mentioned.*

**Response #3-2:** We sincerely appreciate your thoughtful feedback. In response to your comments, we have revised the manuscript to address the concerns raised in Reviewer 2’s Comment #2-1. Specifically, we have added a detailed explanation in the Introduction section (lines 74–84) to clarify the rationale for investigating the psychological effects of mask-wearing in this study. In addition, we have incorporated references that support the validity and reliability of the GHQ-12, which was used to assess psychological distress. These studies indicate that the GHQ-12 is a well-validated instrument that has been applied across diverse cultural and clinical contexts, including various mental disorders (Methods section, lines 152–155).

“Wearing a mask has been shown to cause physical discomfort, such as difficulty breathing, ear pain, and headaches [12], which may influence emotional states and lead to psychological distress. At the same time, wearing a mask may provide a sense of protection, which can help reduce psychological stress [13]. During the COVID-19 pandemic, mask-wearing was recommended to prevent infection. People were expected to adjust their behavior depending on various conditions, including whether they lived in urban or rural areas, the infection status in their surroundings, and whether they were indoors or outdoors. Amid the intense media coverage, vulnerable individuals such as those with mental disorders may have experienced increased psychological stress due to heightened anxiety and greater sensitivity to how others perceived their mask-wearing behavior.”

“The GHQ-12 has shown its effectiveness across various population groups, cultures, and languages [19, 20] as a simple, rapid and comprehensive screening tool for psychological distress and well-being. It has been used in studies targeting psychiatric patients with depression and anxiety disorders [21, 22].”

“19.	Habibi Asgarabad M, Etesam F, Salehi Yegaei P, Vahabi Z, Akbari Saneh N, Fathi F, et al. Construct validity of the General Health Questionnaire (GHQ-12) in patients with COVID-19 and its demographic and medical correlates. Front Psychol. 2023;14: 1132154.

20.	Wojujutari AK, Idemudia ES, Ugwu LE. The evaluation of the General Health Questionnaire (GHQ-12) reliability generalization: A meta-analysis. PLoS One. 2024;19: e0304182.

21.	Pal R, Sachdeva N, Bhansali A, Sharma A, Walia R. Prevalence of Depression and Psychological Distress and Perturbations of Cortisol Dynamics in Attendants of Hospitalized Patients: An Observational Pilot Study. Cureus. 2020;12: e12067.

22.	Bandyopadhyay G, Sinha S, Sen B, Sen G. Validity of General Health Questionnaire (GHQ-36/GHQ-12) in the psychiatric O.P.D. of a general hospital--a pilot study. Int J Soc Psychiatry. 1988;34: 130-134.”

**Comment #3-3:** *Also mention about the management of biases in this study that might have impacted in the study findings.*

**Response #3-3:** Thank you for highlighting this important point. We revised the entire Limitation section (lines 359–374) to clearly acknowledge and discuss potential sources of bias, including selection bias, recall bias due to self-reported data, and unmeasured confounding factors such as medication changes and external events. To improve clarity, we also reorganized the Limitation section to present each potential bias in a structured manner. We believe these revisions enhance the transparency and robustness of our study.

“This study has several limitations that deserve consideration. First, the study was conducted at a single center in rural Japan, which may introduce selection bias and limit the generalizability of the findings to other regions or populations. However, mask-wearing rates were found to be consistent nationwide, and the homogeneity of the study population may also serve as a strength in controlling for regional variability. Second, the assessment of mask-wearing behavior relied on self-reported data, which may be subject to recall bias. Although participants were asked about recent and clearly defined behaviors, some degree of misreporting cannot be ruled out. Third, unmeasured confounding factors such as ongoing treatment effects, changes in medication, seasonal influences, and external events (e.g., national celebrations, including Japan’s victory in the World Baseball Classic or other social events) may have impacted the outcomes. While we included baseline GHQ-12 scores as a covariate to partially account for individual differences in psychological distress, residual confounding may still exist. Lastly, the relatively small sample size limited our ability to conduct subgroup analyses by diagnosis or to examine long-term effects. Future multi-center studies with larger and more diverse populations, as well as longitudinal designs, are needed to confirm and expand upon these findings.”

We have revised our manuscript, including all the requested changes by the reviewers. We believe that our revised manuscript is now suitable for publication in *PLOS ONE.* We look forward to hearing from you regarding our submission. We would be glad to respond to any further questions and comments should you have any.

Sincerely,

Kazuhiro Suzuki, MD, PhD

Department of Community Mental Health

Shinshu University School of Medicine

3-1-1, Matsumoto, Nagano, Japan,

Tel: +81-263-372638

Fax: +81-263-361772

E-mail: kazsuzuki@shinshu-u.ac.jp
